# Mechanisms of Atomic Oxygen Erosion in Fluorinated Polyimides Investigated by Molecular Dynamics Simulations

**DOI:** 10.3390/molecules29184485

**Published:** 2024-09-21

**Authors:** Shengrui Zhou, Li Zhang, Liang Zou, Bilal Iqbal Ayubi, Yiwei Wang

**Affiliations:** School of Electrical Engineering, Shandong University, Jinan 250061, China

**Keywords:** colorless polyimide, low earth orbit, atomic oxygen, molecular dynamics

## Abstract

Traditional polyimides have highly conjugated structures, causing significant coloration under visible light. Fluorinated colorless polyimides, known for their light weight and excellent optical properties, are considered ideal for future aerospace optical lenses. However, their lifespan in low Earth orbit is severely limited by high-density atomic oxygen (AO) erosion, and the degradation behavior of fluorinated polyimides under AO exposure is not well understood. This study uses reactive molecular dynamics simulations to model two fluorinated polyimides, PMDA-TFMB and 6FDA-TFMB, with different fluorine contents, to explore their degradation mechanisms under varying AO concentrations. The results indicate that 6FDA-TFMB has slightly better resistance to erosion than PMDA-TFMB, mainly due to the enhanced chemical stability from its -CF_3_ groups. As AO concentration increases, widespread degradation of the polyimides occurs, with AO-induced cleavage and temperature-driven pyrolysis happening simultaneously, producing CO and OH as the main degradation products. This study uncovers the molecular-level degradation mechanisms of fluorinated polyimides, offering new insights for the design of AO erosion protection systems.

## 1. Introduction

Since the 1960s, polyimides (PIs) have been widely used in aerospace due to their lightweight properties and excellent mechanical and dielectric performance [1,2,3,4,5]. However, traditional aromatic polyimides feature highly conjugated molecular structures that tend to form charge-transfer complexes, resulting in yellow or brown films, which limits their optical applications [6,7]. Introducing fluorine-containing groups into polyimides has been shown to effectively reduce charge-transfer complex formation and enhance thermal conductivity, making fluorinated polyimides suitable for aerospace optical systems [8,9,10,11].

Research into improving the optical, dielectric, and thermal stability properties of polyimides through fluorine substitution is ongoing. He et al. demonstrated that fluorine content significantly impacts the dielectric constant, with different fluorine substituents affecting the constant depending on their position and structure [12]. Zuo et al. developed a highly transparent, low-dielectric-constant colorless polyimide by introducing meta-substituted structures and trifluoromethyl groups, resulting in films with excellent optical transparency and good thermal and mechanical properties [13]. Zhu et al. synthesized polyimide films containing trifluoromethyl groups through high-temperature polycondensation, finding that ortho-trifluoromethyl groups confer exceptional thermal stability by inhibiting the rotation between imide and benzene rings, thus reducing charge-transfer complex formation [14]. Shi et al. explored the properties of fluorinated copolyimide films, revealing that copolymers exhibit superior thermal stability, optical transparency, solubility, and gas permeability [15]. Barra et al., in their review, explored the potential of fluorinated polyimides for space applications. They found that introducing fluorine-containing groups significantly improves the optical transparency and processability of polyimides, making them suitable for solar cell encapsulation in low Earth orbit (LEO) spacecraft and other radiation-resistant aerospace materials [16].

In LEO, spacecraft materials degrade due to exposure to high-energy atomic oxygen (AO), ultraviolet radiation, and ionizing radiation, significantly affecting their operational lifespan [17,18]. Among these factors, AO, with its high density (10^15^ atoms cm^−2^s^−1^) and kinetic energy (4–5 eV), is the primary threat to spacecraft [19,20,21,22,23]. Ground-based AO simulation experiments by Hu et al. showed that polyimide surfaces become significantly rougher and exhibit deteriorated optical properties after AO erosion [24]. Zhang et al. observed that fluorinated colorless polyimide films, initially smooth, become rougher and less transparent to visible light upon AO exposure. Shu et al. found that AO erosion leads to the reformation of charge-transfer complexes in polyimides, deepening the film color, reducing transmittance, and causing a red shift in the UV cutoff wavelength [25]. Shimamura et al. investigated the degradation of mechanical properties in polyimide films exposed to AO in LEO. They found that AO irradiation significantly reduced the tensile strength and elongation of the films, along with decreases in mass and thickness, which severely limits their use in deployable spacecraft structures [26].

Ground exposure experiments are useful for studying polyimide degradation under AO erosion; however, the detailed reaction pathways and intermolecular interactions between polyimides and AO remain unclear [27,28,29]. Reactive force field (ReaxFF) molecular dynamics (MD) simulations have advanced the understanding of AO erosion mechanisms at the molecular level [30]. Wei et al. used ReaxFF MD simulations to investigate the damage and mechanical degradation of polyimides and their protective coatings under AO erosion [31]. Zeng et al. analyzed the decomposition behavior of polyvinylidene fluoride, perfluoropolyhedral oligomeric silsesquioxane, and their composites under AO impact, assessing temperature changes and mass loss [32]. Jeon et al. examined surface chemical changes and disintegration of various polyimide thermal protection nanocomposites under AO erosion, finding that AO bombardment mainly drives material degradation through oxidation and desorption [33]. Zhang et al. investigated the degradation of fluorinated colorless polyimide films in LEO using ground-based AO exposure experiments and reactive molecular dynamics simulations [34]. However, current simulations of AO erosion lack in-depth explorations of how varying fluorine content affects the anti-AO performance of polyimides, limiting the comprehensive understanding and optimization of fluorinated polyimide materials.

Existing studies have shown that fluorine-containing groups enhance the chemical stability and radiation resistance of materials, but their specific effects under AO erosion are still unclear. This study uses ReaxFF molecular dynamics simulations to systematically examine the resistance of two transparent polyimides, PMDA-TFMB and 6FDA-TFMB, with varying fluorine contents under AO erosion at the atomic level. By analyzing changes in mass, damage extent, temperature, and by-product formation under different AO concentrations, the degradation mechanisms of fluorinated polyimides were identified, highlighting the influence of fluorine content on their resistance in harsh low-Earth-orbit conditions. This research provides theoretical insights into the AO erosion mechanisms of fluorinated polyimides and practical guidance for the design of aerospace materials.

## 2. Results and Discussion

### 2.1. Erosion Kinetics of Fluorinated Polyimides

**Figure 1:**
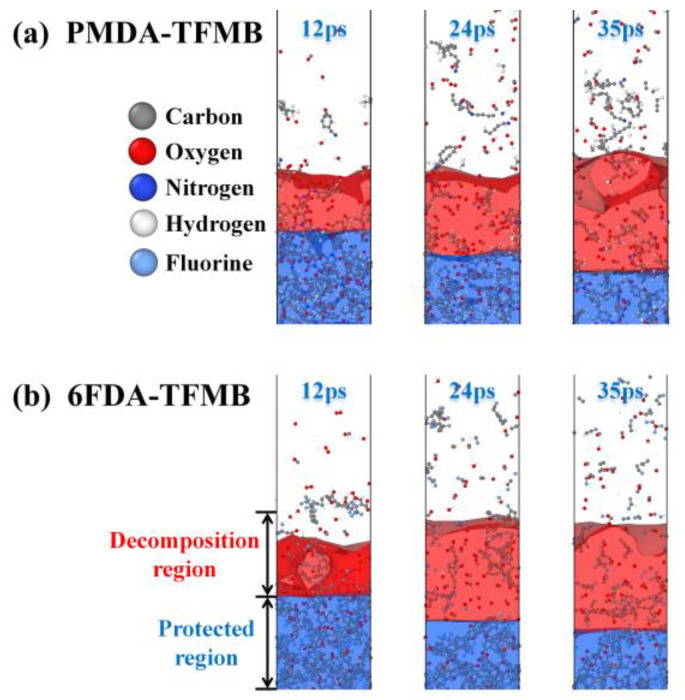
Snapshots of (**a**) PMDA-TFMB and (**b**) 6FDA-TFMB model reactions under AO erosion.

Figure 1 illustrates the structural changes that take place during AO erosion. The region eroded and penetrated by AO is called the decomposition region, while the underlying polymer that AO has not yet reached is the protected region. High-energy AO erosion causes damage in both models, with numerous small molecular fragments escaping from the polymer surface. These fragments may reattach after repeated AO impacts, which may slow further AO erosion, acting as a self-sacrificial defense mechanism. The interface between the decomposition and protected regions continues to recede, expanding the decomposition region. Significant loosening and cracking are observed in the PMDA-TFMB polymer base, attributed to the high reactivity of AO, which reacts with fluorinated polyimides and injects heat into the polymer, causing loosening and cracking as temperatures reach pyrolysis thresholds.

**Figure 2:**
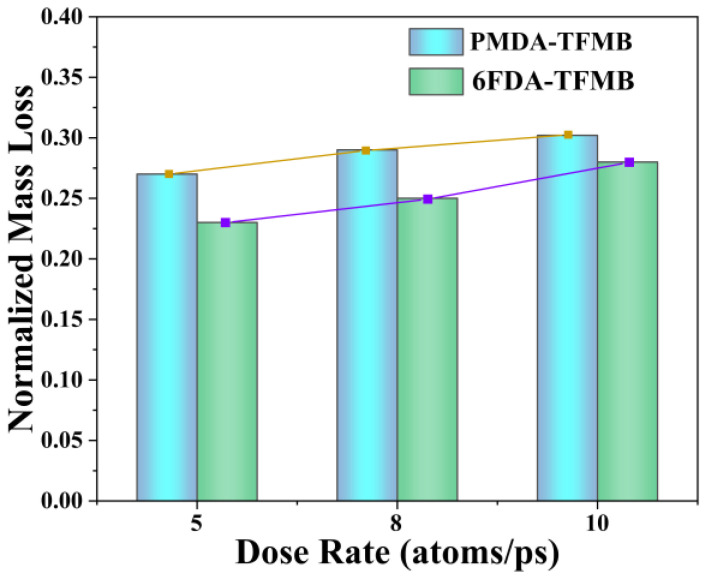
Normalized mass loss of two models under different AO erosion doses.

To quantitatively assess the erosion resistance of the models, the normalized mass loss of the polymer models under various AO doses was calculated. The normalized mass loss, determined by dividing the lost mass by the initial mass, is shown in Figure 2. Across all erosion doses, 6FDA-TFMB exhibits lower mass loss compared to PMDA-TFMB, which can be attributed to the high bond energy of C-F bonds due to fluorine’s strong electronegativity, with more -CF_3_ groups enhancing chemical stability. As AO doses increase, mass loss shows a convergent trend due to increased collision probabilities between AO and small fragments, causing escaped fragments to repeatedly detach and reattach to the polymer surface.

**Figure 3:**
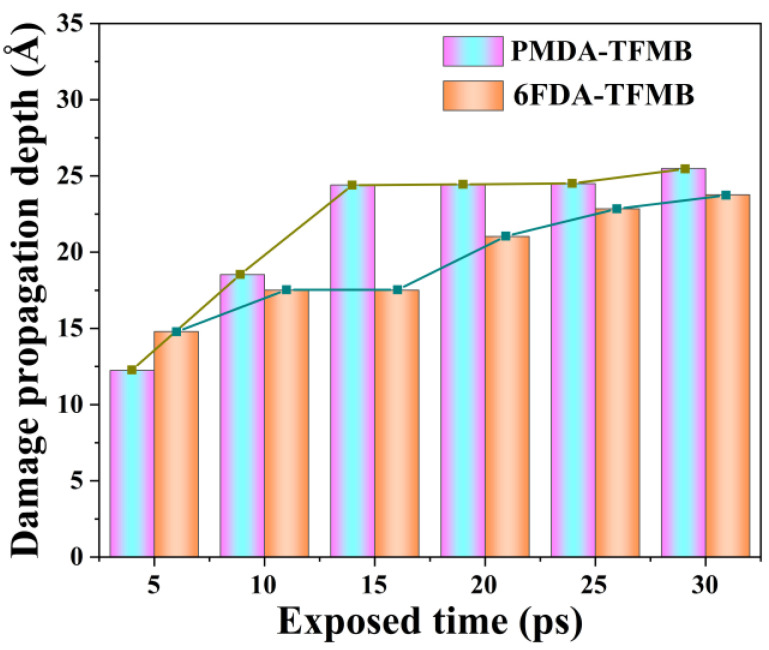
Maximum damage propagation depth during AO erosion.

Figure 3 shows the maximum damage propagation depth during AO erosion simulations. As the reaction progresses, the damage propagation depth of both models increases. During initial AO erosion (first 15 ps), the PMDA-TFMB model shows nearly linear damage propagation, with the polymer undergoing rapid decomposition, reaching maximum damage depth around 15 ps before stabilizing, indicating peak decomposition rates. In contrast, 6FDA-TFMB shows three stages of damage propagation: an initial increase due to AO impact (0–10 ps), a stable phase (10–15 ps), and further increases (25–30 ps) as rising temperatures make the sparse polymer more susceptible to AO penetration, extending damage depth.

### 2.2. Temperature Evolution during AO Erosion

**Figure 4:**
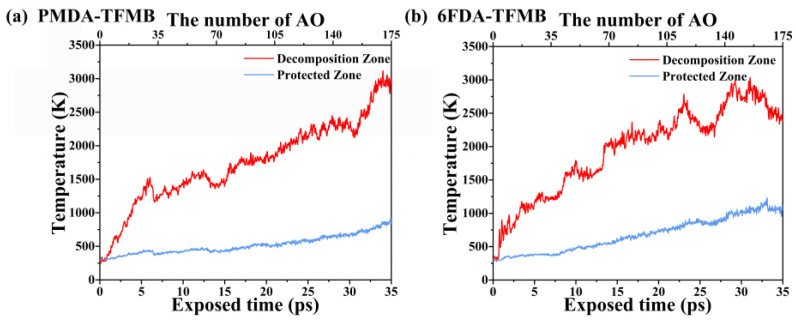
Temperature evolution curves during AO erosion for (**a**) PMDA-TFMB and (**b**) 6FDA-TFMB.

To investigate the temperature evolution of the two fluorinated polyimide models under continuous AO impact, temperature changes in the decomposition and protected regions were calculated, as shown in Figure 4. Both models show similar temperature trends, with sharp increases in the decomposition region directly impacted by AO. After 35 ps of AO erosion, the PMDA-TFMB decomposition region reaches approximately 3000 K. The 6FDA-TFMB model experiences a sharp temperature drop at 23 ps, indicating the pyrolysis threshold is reached, with simultaneous AO impact and polymer pyrolysis causing heat dissipation through escaping small molecular fragments. The temperature peaks at 30 ps before declining again, reflecting a periodic cycle of AO-induced heating and heat dissipation through fragment escape. The protected region heats more slowly due to effective blocking by the polymer above, relying on heat exchange with the decomposition region.

**Figure 5:**
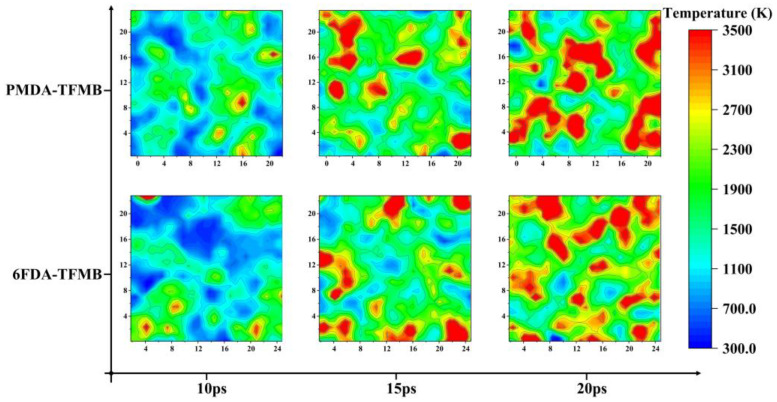
Local temperature distribution near Z = 20 Å for PMDA-TFMB and 6FDA-TFMB at different times.

Further analysis calculated local temperatures near Z = 20 Å at 10 ps, 15 ps, and 20 ps of AO erosion for both models, shown in Figure 5. In early stages (first 10 ps), temperature distributions show no significant differences. By 15 ps, both models develop local hotspots, with AO transferring heat to these regions, and 6FDA-TFMB showing slightly more hotspots due to earlier fragment escape from PMDA-TFMB, causing temporary cooling. At 20 ps, PMDA-TFMB exhibits more concentrated hotspots, with nearby polymers undergoing significant pyrolysis and heat dispersion.

### 2.3. AO Erosion Product Analysis

**Figure 6:**
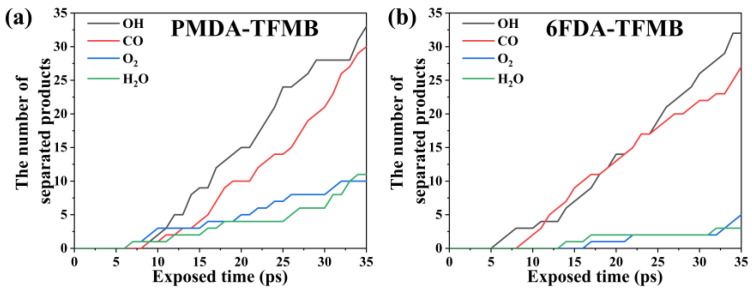
Product counts during AO erosion of (**a**) PMDA-TFMB and (**b**) 6FDA-TFMB.

During AO erosion simulations, types and quantities of small molecular substances separated from the polymer surface above 10 Å were recorded every 1 ps, shown in Figure 6. Primary products include OH, CO, O_2_, and H_2_O. Before 5 ps, no small molecule escape is detected, as AO energy injection has not reached polymer degradation thresholds. As AO erosion continues, OH and CO quantities rise sharply. In reactions between AO and polyimides, OH forms from dehydrogenation reactions between AO and polymers, while CO results from the decomposition of benzene and imide rings. O_2_ forms from AO recombination and collisions with carbonyl groups in imide rings, and H_2_O forms from continuous AO dehydrogenation and collisions between OH and free hydrogen atoms. OH is the most abundant product, followed by CO, making them primary erosion products. 6FDA-TFMB shows slightly lower O_2_ and H_2_O quantities due to the presence of more -CF_3_ groups near imide rings. The strong electron-withdrawing effect of -CF_3_ reduces the electron density of C=O and C-N bonds, making these groups less susceptible to oxidation [35]. Additionally, steric hindrance from -CF_3_ groups prevents AO from attacking C=O bonds, reducing reaction likelihood with AO [36].

**Figure 7:**
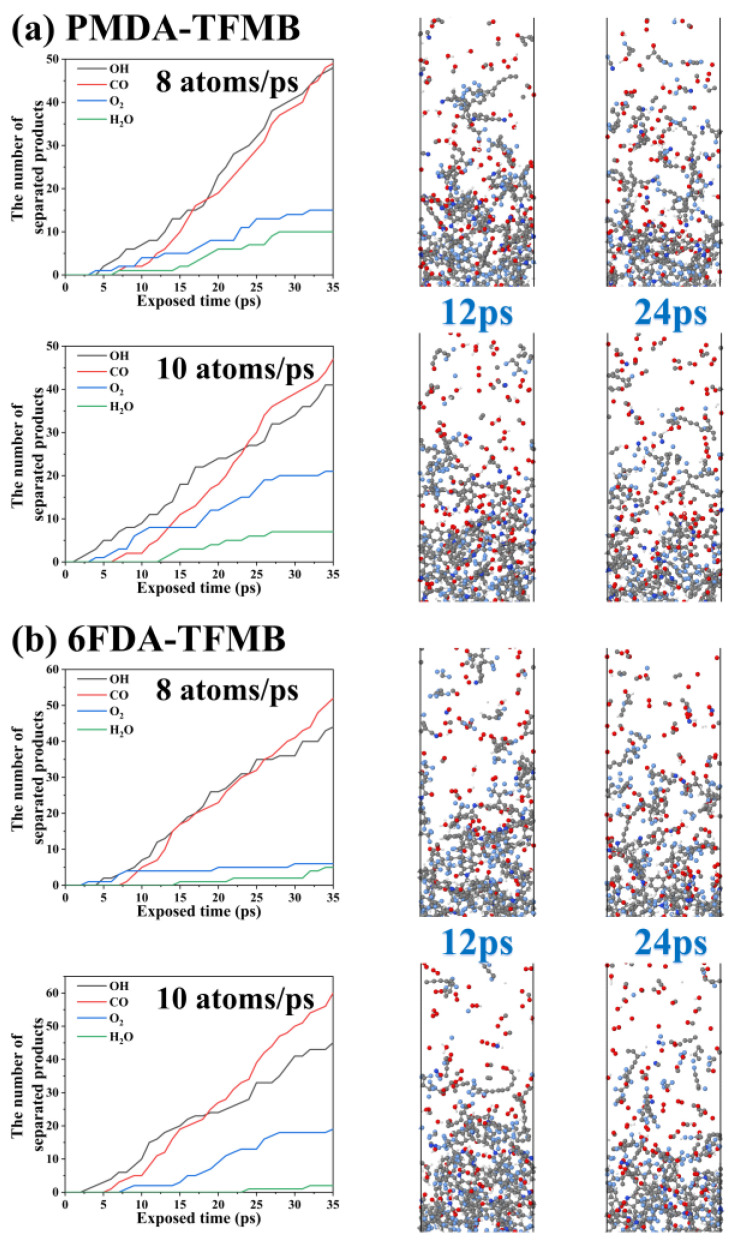
By-product counts and reaction snapshots for (**a**) PMDA-TFMB and (**b**) 6FDA-TFMB under different AO injection frequencies.

To study the impact of different AO erosion concentrations on by-product quantities, PMDA-TFMB and 6FDA-TFMB models were analyzed under AO injection frequencies of 8 atoms/ps and 10 atoms/ps, as shown in Figure 7. Increasing injection frequency raises small molecule product counts, with CO exceeding OH and becoming the primary product. Extensive polymer degradation is observed, with polyimide molecules escaping as carbon chains, and bottom polyimides becoming loose, with simultaneous AO impact and polymer pyrolysis leading to rapid degradation.

**Figure 8:**
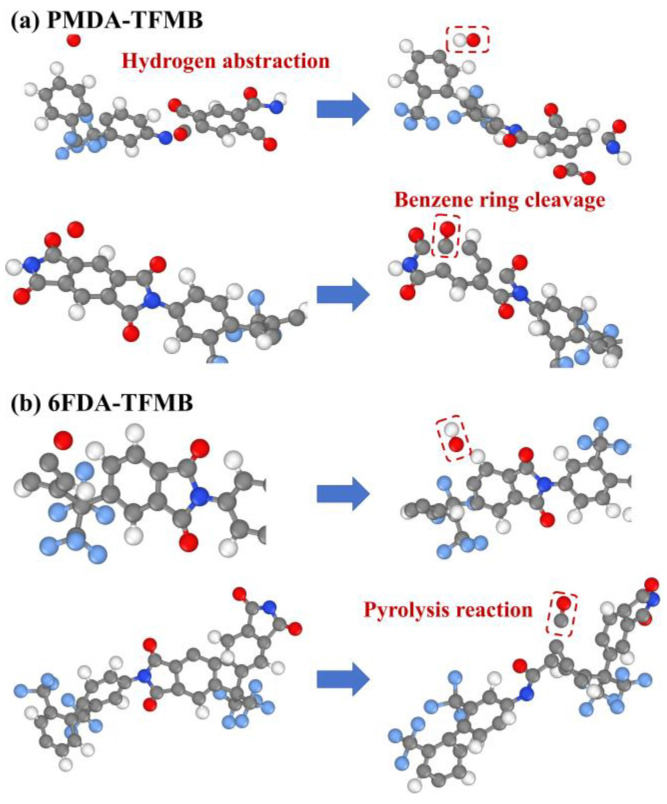
Formation Pathways of Major Products OH and CO in (**a**) PMDA-TFMB and (**b**) 6FDA-TFMB during AO Erosion.

Figure 8 illustrates the primary pathways for OH and CO formation under atomic oxygen (AO) erosion in PMDA-TFMB and 6FDA-TFMB. The high abundance of hydrogen atoms within the polymer system facilitates hydrogen abstraction reactions with AO. In fluorinated polyimides, the -CF_3_ groups significantly withdraw electron density [37], altering the electronic distribution around the benzene rings and polarizing the carbon-hydrogen bonds. This polarization increases the partial positive charge on the hydrogen atoms, making them more susceptible to reacting with AO to form hydroxyl radicals. As erosion progresses, AO reacts with carbon atoms in the polyimide structure, leading to the cleavage of benzene and imide rings and resulting in the production of small molecules such as CO. Additionally, AO continuously transfers its kinetic energy into the system as heat, increasing the polymer temperature and inducing pyrolysis of the polyimides [38]. The combined effects of AO impact and thermal decomposition accelerate CO formation.

## 3. Methods and Models

Reactive molecular dynamics simulations of AO erosion were conducted using the Reax module in LAMMPS [39]. The ReaxFF force field updates and evaluates bond lengths, bond orders, and the relationship between these bond orders and bond energies at each MD simulation step, allowing for continuous bond formation and breakage [40,41]. To enhance computational efficiency, the method partially neglects atomic internal electron transfer and relativistic effects, while still accounting for intermolecular interactions, including van der Waals and Coulomb forces.

**Figure 9:**
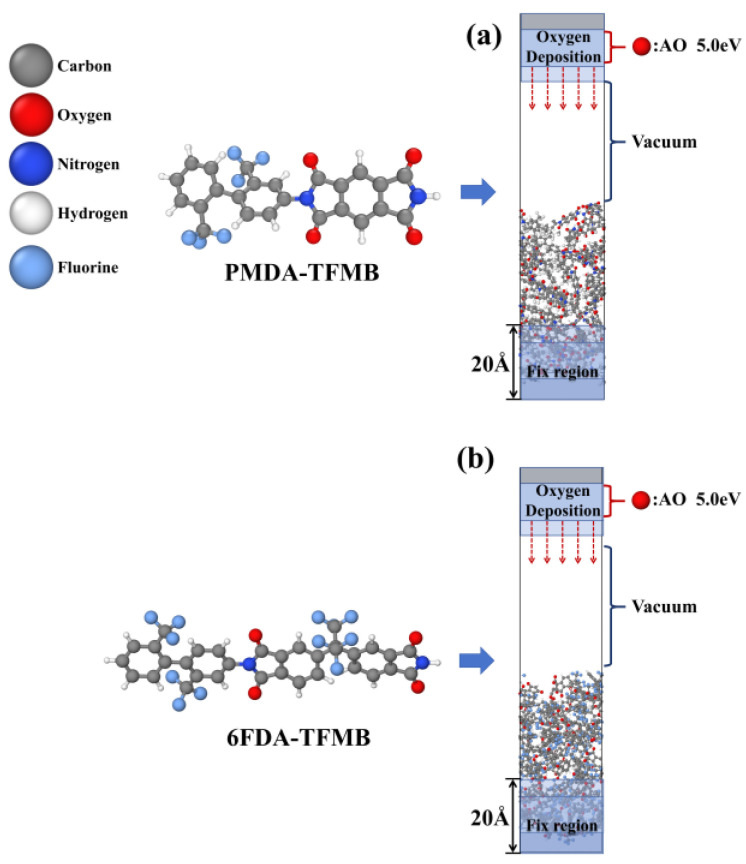
Configurations and crystal structures of (**a**) PMDA-TFMB and (**b**) 6FDA-TFMB.

Figure 9 presents the molecular configurations and equilibrium crystal structures of PMDA-TFMB and 6FDA-TFMB used in this study. Each of the 36 PMDA-TFMB and 25 6FDA-TFMB polyimide chains were placed into a periodic unit cell of 20 Å × 20 Å × 60 Å, followed by a 500 ps relaxation in the NPT ensemble at 298 K and 1 atm. The cell densities stabilized at equilibrium values (ρ_PMDA-TFMB_ = 1.47 g/cm^3^, ρ_6FDA-TFMB_ = 1.46 g/cm^3^), closely matching the standard densities (1.49 g/cm^3^ for PMDA-TFMB and 1.48 g/cm^3^ for 6FDA-TFMB). After relaxation, the cell sizes were 23.17 Å × 23.17 Å × 38.97 Å for PMDA-TFMB and 22.99 Å × 22.99 Å × 39.18 Å for 6FDA-TFMB. The periodic boundary in the Z direction was removed and extended to 120 Å, followed by an NVT ensemble relaxation at 300 K for 500 ps. To prevent model displacement during AO impact, the bottom 20 Å of atoms were fixed as a substrate buffer layer.

To simulate AO erosion effects at different concentrations, AO with kinetic energy of 5 eV [42,43] was inserted 50 Å above the model surface at rates of 5 atoms/ps, 8 atoms/ps, and 10 atoms/ps, with a simulation timestep of 0.1 fs for 35 ps. All AO erosion simulations were performed in the NVE ensemble to visually observe surface structural changes. The force field parameters used were developed by Rahnamoun et al. [44].

## 4. Conclusions

This study uses reactive molecular dynamics simulations to model PMDA-TFMB and 6FDA-TFMB fluorinated polyimides, investigating their degradation processes, mass loss, damage propagation, temperature evolution, and erosion by-products under AO erosion in LEO at varying concentrations. The following conclusions were drawn:

Fluorinated polyimides gradually degrade under AO erosion, with the interface between decomposition and protected regions descending continuously. 6FDA-TFMB exhibits better chemical stability due to higher fluorine content. As AO injection increases, erosion reaches saturation, and changes in normalized mass loss are minor. Continued reactions cause the polymer to become sparse, allowing AO to penetrate further, deepening maximum damage propagation.

During AO erosion, decomposition region temperatures rise sharply, with heat dissipation through small fragment release, while protected regions heat through exchange with decomposition zones. Persistent AO erosion generates numerous localized hotspots in bottom polyimides.

Continuous AO impact generates substantial small gaseous by-products from PMDA-TFMB and 6FDA-TFMB, increasing with higher erosion concentrations. OH and CO dominate the by-products, reflecting dehydrogenation and carbon chain break reactions between AO and polyimides.

This study systematically analyzed the degradation process of fluorinated polyimides under atomic oxygen erosion at the atomic scale, offering new insights into their corrosion resistance in low Earth orbit. It was found that 6FDA-TFMB, with its higher fluorine content and the electron-withdrawing effect of its -CF_3_ groups, demonstrated superior resistance to AO erosion. By revealing the primary degradation pathways and product formation mechanisms under AO exposure, this research provides theoretical support for selecting AO-resistant materials for spacecraft in low Earth orbit and offers guidance for the molecular design and optimization of fluorinated polyimides.

## Data Availability

The data presented in this study are available on request from the corresponding author.
